# Four Cases of Placenta Previa

**Published:** 1899-03

**Authors:** O. C. Hammond

**Affiliations:** Fine, St. Lawrence Co., N. Y.


### CORRESPONDENCE.

### FOUR CASES OF PLACENTA PREVIA.

FINE, ST. LAWRENCE CO., N. Y.

*Editor of Buffalo Medical Journal:*

*Sir.*—The following account of four cases of placenta previa occurring in the work of a general practitioner, within a space of five months, may prove interesting on account of the infrequency of such a series of events.

My first case was one in which the placenta partially overlapped the dilated os, placenta previa partialis, a segment of the placenta being distinctly recognisable and also a portion of the membranes. In this case there was considerable hemorrhage at the end of the eighth month, coming on without pain and in the night. This case was allowed to progress to full term, and the woman was finally delivered of a living child by turning and extraction by the feet. My consultant in this case was Dr. B. F. Drury, of Gouverneur, N. Y., who did not arrive, however, until after the child was born. Both mother and child did well.

My next case, which occurred the following month, did not terminate so favorably. This case was one of complete central implantation, the placenta wholly covering the dilated os. Dr. Drury was my consultant at this time. This mother was almost completely exhausted by hemorrhage at the time I was called and owing to difficulties encountered in attempting version, we were compelled to deliver with forceps. The child was born dead and the mother died in about an hour afterward from the effects of the previous hemorrhage, she having lost but little blood during the operation.

My next case occurred about two months after the second and here also the os was completely covered by the placenta. This mother had frequent and oft-recurring hemorrhages, beginning as early as the seventh month. I allowed her, however, to go to term and then delivered her by turning and immediate extraction. I was assisted in this case by Dr. Taylor, of Edwards, N. Y. Both mother and child lived, but the child died not long afterward. My fourth and last case occurred about five months from the first. This was one in which the os was but partially overlapped. The mother was a strong and healthy young woman. Delivery in her case was easily performed by podalic version, nature being allowed to complete the case as the hemorrhage was inconsiderable. Both mother and child lived. With this last case as with the first, I was alone, being far in the country and no other physician in reach.

O. C. HAMMOND, M. D.

January 9, 1899.

